# Supplementary material for: The causal role of circulating inflammatory markers in osteoporosis: a bidirectional Mendelian randomized study
Source: Front Immunol. 2024 Jul 18;15:1412298. doi: 10.3389/fimmu.2024.1412298 (PMC11291241; doi:10.3389/fimmu.2024.1412298)
Supplement: Supplementary file 1 [file DataSheet_1.zip › supplementary material/CIM-OSTEOPOROSIS /GCST90274830.csv_forest.pdf]

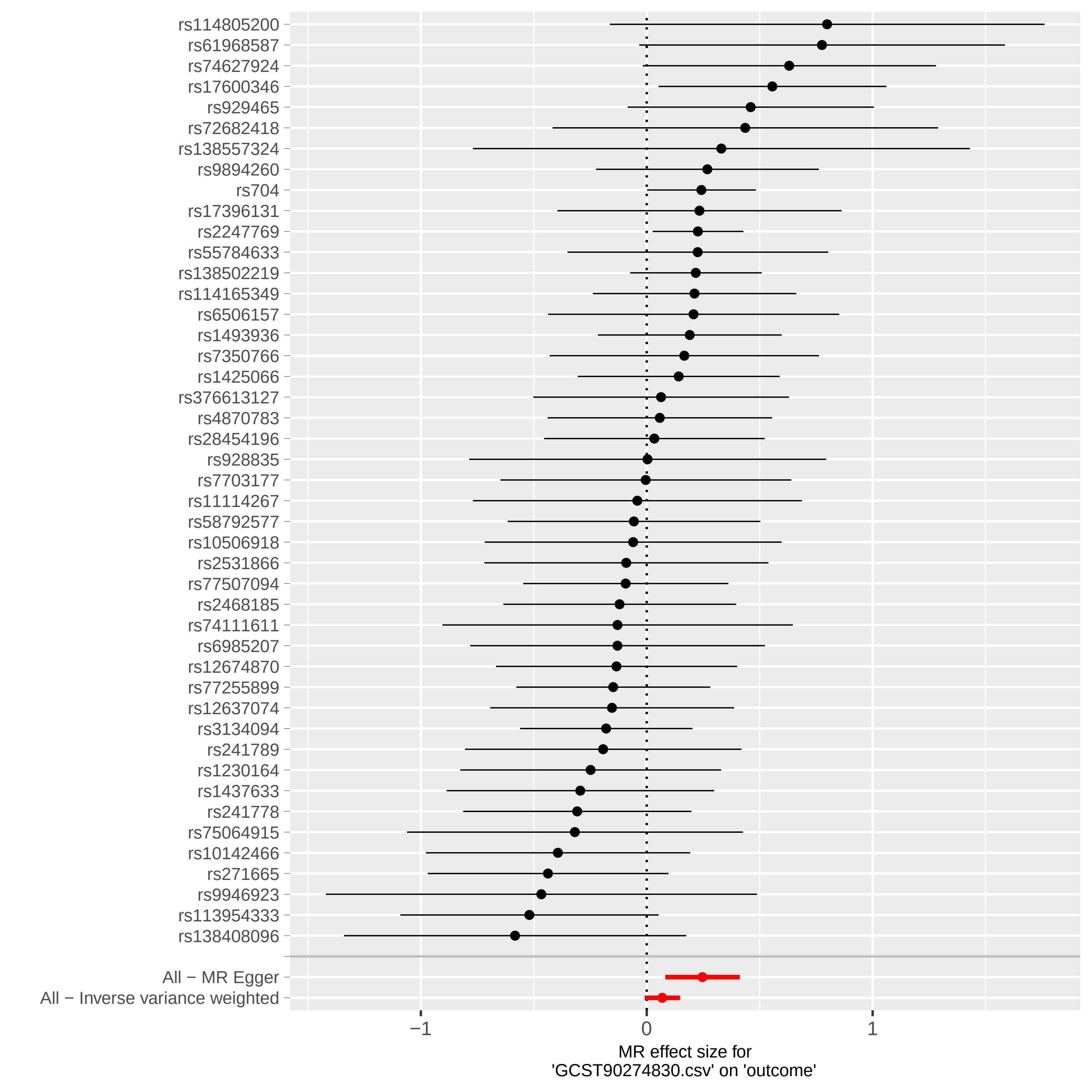
rs114805200
rs61968587
rs74627924
rs17600346
rs929465
rs72682418
rs138557324
rs9894260
rs704
rs17396131
rs2247769
rs55784633
rs138502219
rs114165349
rs6506157
rs1493936
rs7350766
rs1425066
rs376613127
rs4870783
rs28454196
rs928835
rs7703177
rs11114267
rs58792577
rs10506918
rs2531866
rs77507094
rs2468185
rs74111611
rs6985207
rs12674870
rs77255899
rs12637074
rs3134094
rs241789
rs1230164
rs1437633
rs241778
rs75064915
rs10142466
rs271665
rs9946923
rs113954333
rs138408096
All − MR Egger
All − Inverse variance weighted
−1
0
1
MR effect size for
'GCST90274830.csv' on 'outcome'
